# Effect of the lack of primary stability in the survival of dental implants

**DOI:** 10.4317/jced.54441

**Published:** 2018-01-01

**Authors:** Carlos Cobo-Vázquez, David Reininger, Pedro Molinero-Mourelle, José González-Serrano, Blanca Guisado-Moya, Juan López-Quiles

**Affiliations:** 1 DDS, MS, Clinical Assistant Professor, Department of Medicine and Oral Surgery, Faculty of Dentistry, Complutense University of Madrid; 2 DDS, MS, PhD Student, Department of Orofacial Prosthetics. Faculty of Dentistry, Complutense University of Madrid; 3 DDS, MS, Master’s Student, Department of Medicine and Oral Surgery, Faculty of Dentistry, Complutense University of Madrid; 4 MD, DDS, PhD, Professor of Oral Surgery, Department of Medicine and Oral Surgery, Faculty of Dentistry, Complutense University of Madrid

## Abstract

**Background:**

The survival of dental implants has been linked to primary stability. The aim of this study is to analyse the factors that influence the survival of dental implants placed without primary stability.

**Material and Methods:**

A cohort study of implants placed without primary stability was carried out between September 2011 and July 2016. All cases with registered information on the patient and surgical intervention were used. Cases that did not have a 12-month follow-up after implant placement were excluded.

**Results:**

Out of 2,400 analysed implants, 92 were placed without primary stability. The absence of primary stability was classified as B in 49 cases, C in 38 cases and D in 5 cases. No statistically significant influence of the patient’s age, primary stability, brand, or implant size in terms of implant survival was established. A tendency towards greater early implant loss was observed in implants whose absence of primary stability was classified as C.

**Conclusions:**

Poor primary stability is not statistically significant in the loss of dental implants of the characteristics studied. Any of the factors studied are related with early implant loss as a main factor.

** Key words:**Primary stability, survival, dental implants.

## Introduction

Long-term success rates of rehabilitation with dental implants have been extensively documented over the past three decades (1-3).

Implant stability has been recognised as one of the most important and useful factors when it comes to predicting implant anchorage. Primary implant stability is defined as the biomechanical stability upon implant insertion, being influenced by numerous factors, such as: bone quantity and quality, the geometric design of the implant, surgical technique, and insertion torque. From this stability, new bone develops around the surface of the implant, constituting a biological fixation named secondary implant stability (4-6).

Although numerous studies describe different techniques used to assess stability upon implant placement or once the osseointegration period has passed, there is controversy related to their accuracy. One of the most popular digital methods is resonance frequency analysis (RFA), Osstell® system (Osstell AB Stampgatan, Gotemborg, Sweden) and Periotest.® (Siemens Medical Systems Inc, Charlotte, Nc). Neither seem to be optimal methods to measure stability nor define success upon implant placement. Regarding clinical methods, measuring insertion torque prevails over others, and as a method of stratifying it, an article published by Rodrigo *et al.* proposes four measurement parameters (6-8).

In terms of survival and stability, different factors such as bone length, diameter or bone quality have been conventionally analysed, but there are few studies that evaluate the absence of primary stability associated with implant treatment success (6,9,10).

The aim of this study is to analyse the factors that influence the survival of dental implants placed with a lack of primary stability.

## Material and Methods

A cohort study of dental implants placed without primary stability was carried out at the Master of Oral Surgery and Implantology of the Faculty of Dentistry of Complutense University of Madrid.

A clinical trial protocol based on the PICO model was established to meet the objective of the study. Patients: Patients with dental implants placed, Intervention: Dental implants without primary stability, Comparison: General factors (sex, tobacco, diabetes); Biological factors (bone resorption, bone quality, vascularization at alveolar ridge, previous bone regeneration); Implant related factors (size, type, manufacturer); Results: Dental implants survival

The following inclusion criteria were established: cases of dental implants placed between September 2011 and July 2016, with a minimum follow-up period of 12 months, where the surgical report shows an absence of primary stability.

As exclusion criteria were: cases where all of the clinical and surgical information was incomplete or not accurate enough, cases that were part of another clinical study, cases that were not followed appropriately because of patient desertion, cases where the recommended surgical drilling sequence for each implant according to the manufacturer was not carried out, patients with a medical history of irradiation to the head and neck region, with uncontrolled diabetes, with uncontrolled periodontal disease, and other mucosal diseases.

The implants were placed following the protocol of the Master of Oral Surgery and Implantology, which includes, medical history, casts, and a complete radiographic study.

In all cases, a Cone Beam Computerised Tomography (CBCT) was performed prior to implant surgery, which was also used to classify bone resorption in: good (B), extensive (C), moderate (D), and poor (E) according to Lekholm and Zarb classification (11). The conventional surgical technique of flap elevation, drilling sequence indicated by each manufacturer, submerged implant placement or non-submerged implant placement according to the implant design, and flap suture was carried out. No cases of postextraction implant placement were included. In no cases was placed a healing abutmentdrilling, and no inmediate crown was placed.

Patients were recalled 7 days after implant placement for suture removal, and monthly thereafter to evaluate osseointegration. In all cases, the prosthodontic phase commenced 3 months after implant placement, with check-ups at 15 days and 30 days following rehabilitation. Radiographic controls were performed upon implant placement, at the time of prosthetic loading, and annually thereafter. All subjects signed an informed consent form prior to surgery, providing their approval to use the information obtained in clinical trials anonymously. No preoperative medication was prescribed. The pharmacological regimen in all cases was amoxicillin at a dose of 750mg (for 8 days), ibuprofen at a dose of 600mg (for 3 days), paracetamol at a dose of 1000mg (in the case of pain, alternating) and omeprazol at a dose of 20mg (for 8 days).

Implant primary stability was classified following the clinical criteria proposed by Rodrigo *et al.* (8), who categorise primary stability absence as the following:

(B) When there is a light rotation with a feeling of resistance;

(C) When the implant rotates without resistance;

(D) When there is both rotation and lateral oscillation of the implant.

The following variables were included: patient age, gender, smoking status, ASA classification antibiotics, bone grafts, residual bone height, implantation region, and implant adjacency, implant length and diameter, and implant design.

Once all the information was recorded, a descriptive statistical analysis of the quantitative variables was performed for the description of the samples. The descriptive statistics of the qualitative variables were studied (FREQUENCIES procedure), resulting in the frequencies and percentages of the categories.

The Kolmogorov-Smirnov Test for a sample (NPAR TESTS procedure) was used to determine if the quantitative variables of the study derive from a normal distribution.

To observe the relationship between qualitative variables, contingency tables (CROSSTABS procedure) were used. An Exact Fisher’s Test or Chi-Square Test was applied to compare the independence or influence between two qualitative variables. To compare the means, the ANOVA test was used (ONEWAY procedure).

As for the comparison of the means of the quantitative variables, Student’s t-Test (T-TEST procedure) was applied.

The statistical analysis of the data was carried out with the SPSS 22.0 (IBM SPSS, 2013) program for Windows.

## Results

-Descriptive study

The study included 2,400 implants placed between 2009 and 2014. Excluding implants placed with primary stability and those which information about surgical procedure was not accurate enough, it was obtained a final sample of 92 implants placed with absence of primary stability.

Out of the 92 implants, 55 (59.8%) were placed in women and 37 (40.2%) in men, where 12 (13%) were smokers against 80 (87%) non-smokers. Only one patient had diabetes (1.1%), and the remaining 91 (98.9%) patients were healthy. As for bone quality, 20 (21.7%) patients presented cortical bone (type I Lekholm and Zarb), 21 (22.8%) presented cortical and porous bone (type II Lekholm and Zarb), and 51 (55.4%) cancellous bone (type IV Lekholm and Zarb). Bone quantity was good (B, Lekholm and Zarb, 1985) in 20 (21.7%), extensive (C, Lekholm and Zarb, 1985) in 1 (1.1%), moderate (D, Lekholm and Zarb, 1985) in 32 (34.8%), and poor (E, Lekholm and Zarb, 1985) in 2 (2.2%). Vascularization was good in 57 (62%), poor in 1 (1.1%), moderate in 32 (34.8%), and poor in 2 (2.2%). Finally, 23 (25%) received bone regeneration, against 69 (75%) who did not ( Table 1).

**Table 1:**
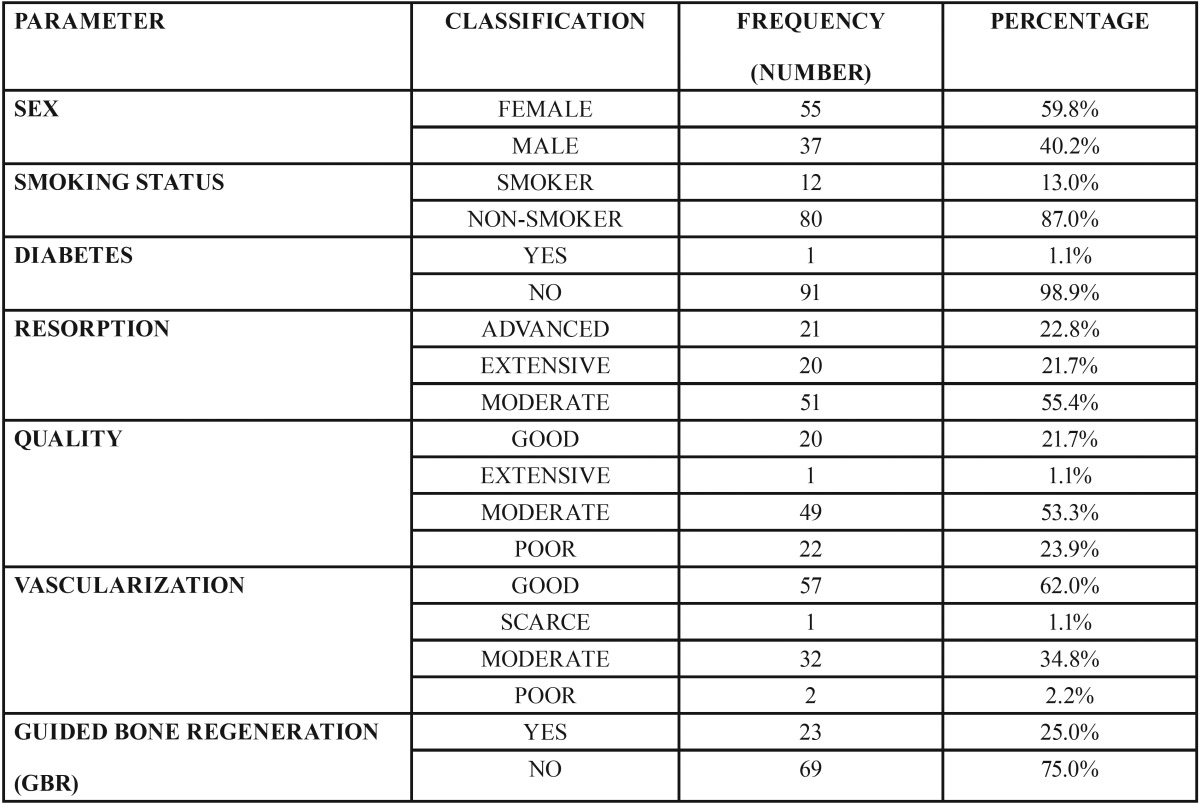
Descriptive statistics of the variables. The distribution of the registered cases according to the following variables: sex, smoking, diabetes, bone resorption, bone quality, vascularization, bone regeneration.

-Primary stability

There were 49 implants (53.26%) with type B primary stability, 38 (41.30%) with C primary stability and 5 (5.44%) with D primary stability. Out of the 3 groups, only 3 (3.26%) implants were lost, where they belonged to category C and represented 7.89% of this group ( Table 2).

**Table 2:**
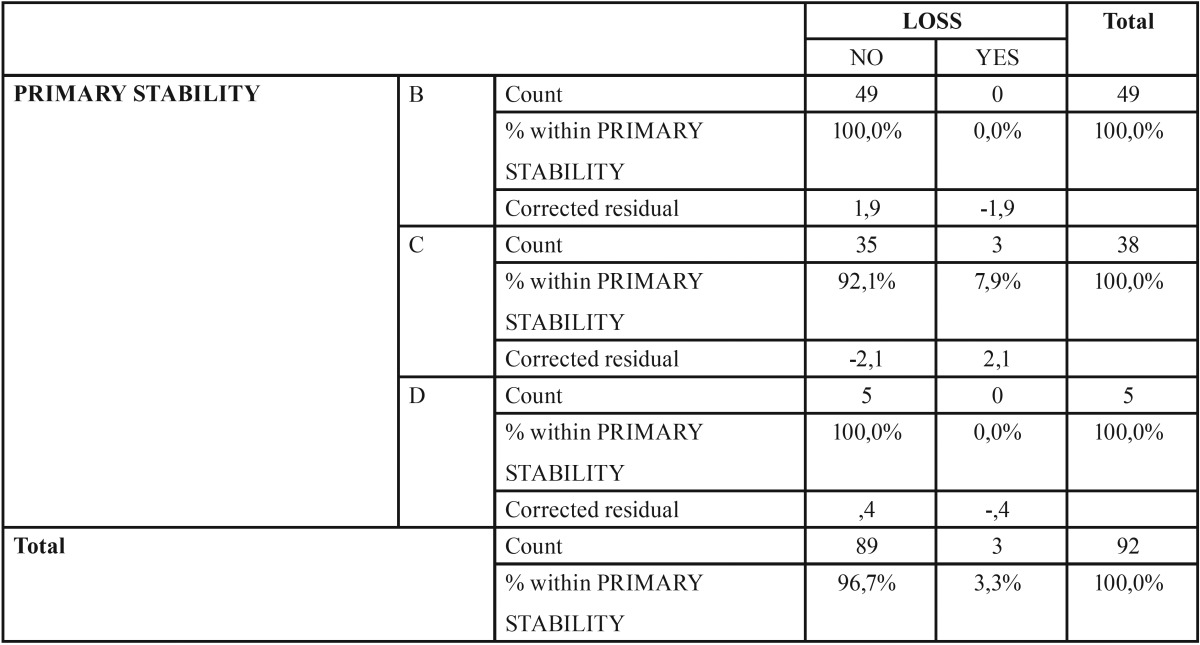
Distribution of implant loss against primary stability. The study of the relationship between the degrees of unachieved primary stability and implant loss is presented.

No statistically significant differences were found, with a 95% confidence interval (*p* = 0.149) in the distribution of early implant loss in the different primary stability groups.

The corrected residual reveals that there is a loss statistically greater than expected in the primary stability category C, being 7.9% against the expected 3.3%.

Implant loss and factor related 

To analyse the existence of any factors related to early implant loss, such as age, implant manufacturer, implant system or size, the statistical analysis was performed using the t-Student test.

In relation to age, there were no statistically significant differences between the primary stability groups (*p* = 0.88), nor implant loss or non-loss (*p* = 0.658). No statistical relationship between implant loss and the manufacturer could be established (*p* = 0.09) with a significance of 95% ( Table 3).

**Table 3:**
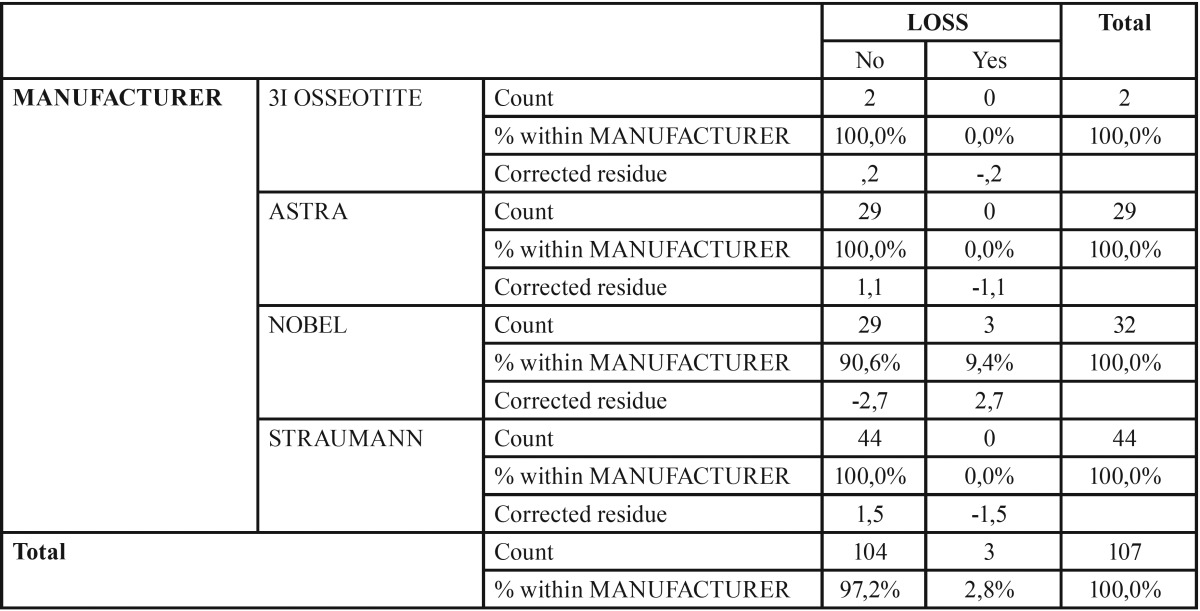
Analysis of the relationship between implant loss and the manufacturer. The study of the relationship between implant loss and the manufacturer is presented. The statistical analysis does not reveal a significant relationship between both variables.

**Figure 1:**
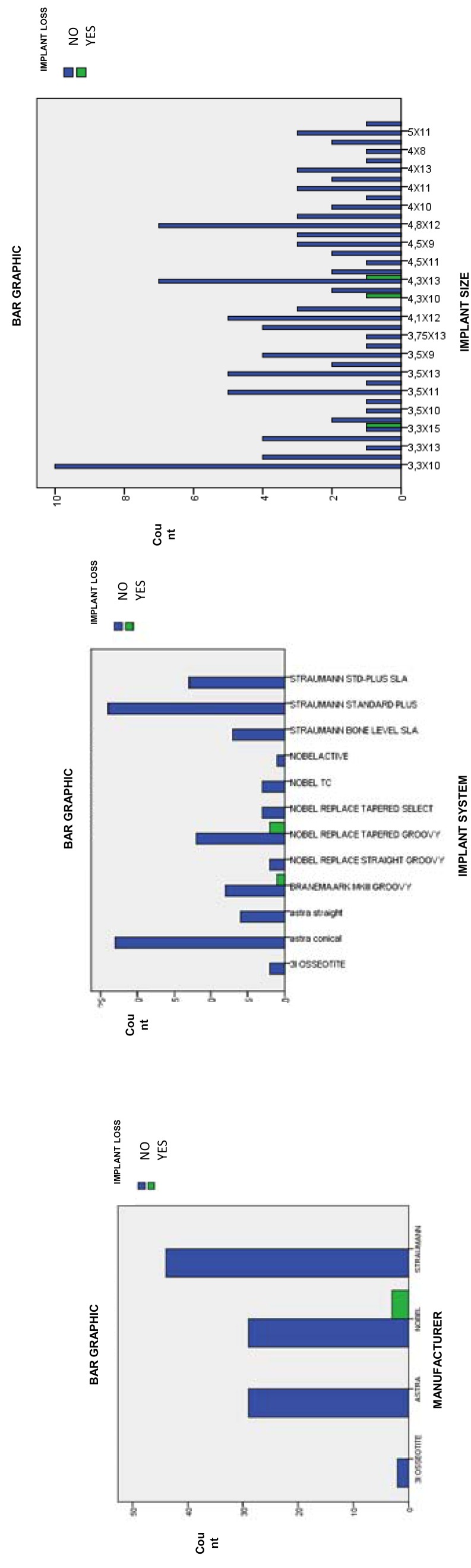
Bar charts: manufacturer, system and implant size in relation to implant loss. The distribution of the placed implants and their losses are presented according to th the manufacturer, their type, and size.

Of the 92 implants placed without primary stability, 2 were 3I OSSEOTITE® (Biomet 3i Dental Iberica, Barcelona, Spain), 29 ASTRA® (Dentsply Sirona, Salzburg, Austria), 29 NOBEL® (NobelBiocare Ibérica SA, Barcelona, Spain) and 44 STRAUMANN® (Institut Straumann AG, Bachel, Switzerland). The 3 that failed were NOBEL implants (Fig. 1), where two were Nobel replace tapered groovy, and one was Branemark MKIII straight groovy. Finally, the size of the analysed implants was between 3 and 5 mm in diameter and between 8-15 mm in length. The lost implants were 4.3x10mm, 4.3x13mm and 3.3x15mm (Fig. 1).

## Discussion

According to our knowledge, the present study is the first to evaluate lack of primary stability during osseointegration in conventional loading. Therefore, the direct comparison of results is unattainable.

The difficulty that lies in quantifying implant survival with poor primary stability should be considered, as at present, there are no standards for their measurement. However, the most used objective methods are resonance frequency analysis (RFA) and the Periotest, frequently debated for their limitations and risks (12,13).

Regarding clinical assessment, we found similarities with Fu *et al.*, who analysed the relationship between bone type according to Leckholm and Zarb, tactile sensation during drilling according to Misch, primary stability measured with an Osstell® device and bone quality analysis by means of computerized microtomography. This study warns of the limitations of primary stability measurement instruments in certain bone densities, especially in the mandible. Therefore, clinical evaluation of implant stability is generally subjective, observational and experience-based (14).

Quesada-García *et al.* analysed diameter, location, and plasma-rich growth factor (PRGF) regarding stability measured by RFA, concluding that narrow implants placed in the maxilla present worse stability when compared to PRGF humidified implants. However, this data is not applicable to ours, since this study did not measure stability during osseointegration (15).

Regarding shape, there is greater primary stability in conical double tapered implants compared to other designs. In compliance with these results, Staedt *et al.* conclude that the greatest primary stability, measured with an Osstell® device, is obtained in cylindrical implants inserted at 30 Ncm, and in conical ones inserted at 40 Ncm. In our study, two of the lost implants had a conical macrodesign, while the third was cylindrical (16,17).

It has been reported that the drilling technique may have a positive effect on primary stability, thus for Falisi it would be with bone expanders, whereas for Degidi, stepped osteotomy (16,18).

In this regard, the results we obtained are not comparable, since conventional milling was carried out following the manufacturer’s instructions.

This study presents a series of limitations regarding its design. The selection of implants without primary stability, the patients’ own conditions, as well as the clinicians’ sensitivity can condition the implants in terms of lack of primary stability.

## Conclusions

Based on the limited sample, poor primary stability is not statistically significant in the loss of implants with the assessed characteristics.

A study with a larger sample is necessary to identify the influence of macrodesign and surface during osseointegration, in situations in which primary stability has not been achieved during surgery.
